# Li-Doped Bioactive Ceramics: Promising Biomaterials for Tissue Engineering and Regenerative Medicine

**DOI:** 10.3390/jfb13040162

**Published:** 2022-09-24

**Authors:** Ahmad Reza Farmani, Mohammad Ali Salmeh, Zahra Golkar, Alaa Moeinzadeh, Farzaneh Farid Ghiasi, Sara Zamani Amirabad, Mohammad Hasan Shoormeij, Forough Mahdavinezhad, Simin Momeni, Fatemeh Moradbeygi, Jafar Ai, John G. Hardy, Amir Mostafaei

**Affiliations:** 1 Tissue Engineering and Applied Cell Sciences Department, School of Advanced Technologies in Medicine, Tehran University of Medical Sciences, Tehran 14166-34793, Iran; 2 Tissue Engineering Department, School of Advanced Technologies in Medicine, Fasa University of Medical Sciences, Fasa 74615-168, Iran; 3 Students’ Scientific Research Center, Tehran University of Medical Sciences, Tehran 14166-34793, Iran; 4 Department of Biotechnology, School of Chemical Engineering, College of Engineering, University of Tehran, Tehran 14155-6619, Iran; 5 Department of Midwifery, Firoozabad Branch, Islamic Azad University, Firoozabad 74715-117, Iran; 6 Department of Tissue Engineering and Regenerative Medicine, Faculty of Advanced Technologies in Medicine, Iran University of Medical Sciences, Tehran 14496-14535, Iran; 7 Cellular and Molecular Research Center, Iran University of Medical Sciences, Tehran 14496-14535, Iran; 8 Department of Chemical Engineering, Faculty of Engineering, Yasouj University, Yasouj 75918-74934, Iran; 9 Emergency Medicine Department, Shariati Hospital, Tehran University of Medical Sciences, Tehran 14166-34793, Iran; 10 Anatomy Department, School of Medicine, Tehran University of Medical Sciences, Tehran 14166-34793, Iran; 11 Department of Infertility, Velayat Hospital, Qazvin University of Medical Sciences, Qazvin 34199-15315, Iran; 12 Chemistry Department, Faculty of Science, Shahid Chamran University of Ahvaz, Ahvaz 83151-61355, Iran; 13 Department of Pharmaceutical Biotechnology, School of Pharmacy, Shiraz University of Medical Sciences, Shiraz 71348-14336, Iran; 14 Pharmaceutical Sciences Research Center, Shiraz University of Medical Sciences, Shiraz 71348-14336, Iran; 15 Department of Chemistry, Faraday Building, Lancaster University, Lancaster LA1 4YB, UK; 16 Materials Science Institute, Lancaster University, Lancaster LA1 4YW, UK; 17 Department of Mechanical, Materials, and Aerospace Engineering, Illinois Institute of Technology, 10 W 32nd Street, Chicago, IL 60616, USA

**Keywords:** lithium, bioceramics, bioactive biomaterials, tissue engineering, cancer treatment, autophagy, drug delivery

## Abstract

Lithium (Li) is a metal with critical therapeutic properties ranging from the treatment of bipolar depression to antibacterial, anticancer, antiviral and pro-regenerative effects. This element can be incorporated into the structure of various biomaterials through the inclusion of Li chloride/carbonate into polymeric matrices or being doped in bioceramics. The biocompatibility and multifunctionality of Li-doped bioceramics present many opportunities for biomedical researchers and clinicians. Li-doped bioceramics (capable of immunomodulation) have been used extensively for bone and tooth regeneration, and they have great potential for cartilage/nerve regeneration, osteochondral repair, and wound healing. The synergistic effect of Li in combination with other anticancer drugs as well as the anticancer properties of Li underline the rationale that bioceramics doped with Li may be impactful in cancer treatments. The role of Li in autophagy may explain its impact in regenerative, antiviral, and anticancer research. The combination of Li-doped bioceramics with polymers can provide new biomaterials with suitable flexibility, especially as bio-ink used in 3D printing for clinical applications of tissue engineering. Such Li-doped biomaterials have significant clinical potential in the foreseeable future.

## 1. Introduction

Tissue engineering and regenerative medicine approaches have a variety of applications in human healthcare, including bone reconstruction and fertility treatments [1,2,3,4,5,6,7,8,9,10]. Tissue engineering combines three components, cells, biomaterials, and bioactive molecules (e.g., drugs and growth factors), to repair the body’s damaged tissues and restore their normal function [11,12,13,14]. Stem cells can self-renew, proliferate, and differentiate into target tissue cells in the presence of relevant growth factors when in biomaterials with supportive architecture, composition, and mechanics [15,16]. However, some studies have shown that stem cells are the cause of pathological conditions and have a role in cancer [17,18,19,20].

**Figure 1:**
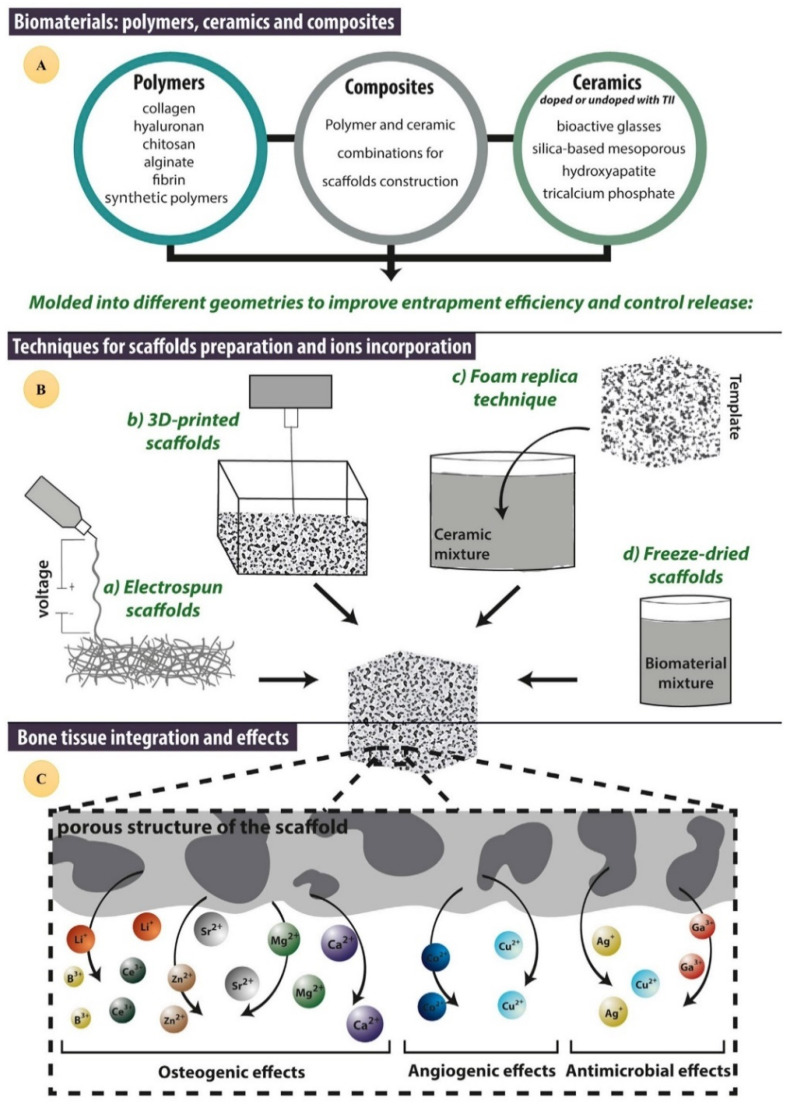
Common loading techniques for therapeutic inorganic ion entrapment in nanoparticles/microparticles, granules, hydrogels, and fibers. Reproduced from [53] with permission.

Biomimetic biomaterials with similar properties to the extracellular matrix play a crucial role in cell proliferation and differentiation, and consequently tissue regeneration. Generally, biomaterials used in tissue engineering can be divided into four main categories: metal-based, ceramic-based, polymer-based, and their composites [21,22,23,24,25,26,27,28,29,30]. Due to their structure and similarity to the extracellular matrix of hard tissues (such as bone and teeth), bioceramics have been widely investigated for their potential application in the regeneration of hard tissues [31,32]; however, by contrast, there has been limited research into their application in soft tissue regeneration [33,34,35], which is in part due to challenges related to engineering bioceramics with mechanical properties mimicking the natural tissues in which they will be implanted [24,36,37,38,39,40,41,42,43,44]. Bioceramic biomaterials can release drugs and/or therapeutic metal ions in regenerative medicine applications and cancer treatment [45,46,47,48,49,50], yet doping the right amount of ions/therapeutics and the controlled/sustained release of the therapeutic payloads are remaining challenges (Figure 1) [51,52,53].

Li is an essential ion that has a multitude of biological effects on the body, such as increasing the activity of chemical messengers in the brain [54,55,56]. The discovery that Li is effective in treating bipolar disorder happened more than 70 years ago, and it is a widely prescribed bipolar disorder medication [57,58,59]. However, it is associated with adverse effects and teratogenicity [60]. Li affects hematopoiesis, embryonic development, glycogen synthesis, and other processes [61,62,63]. Its mechanism of action in mood disorders was unknown in the past; it was thought that Li exerts its effect by affecting cation transport in nerve and muscle cells [64]. In pharmacy, Li is prepared in the form of carbonate, acetate, citrate, sulfate, and orotate salts, and it is most commonly prescribed in the form of carbonate or citrate salts in tablets (up to 2.0 g per day) [55,65]. However, concerns related to Li include hand tremor, downbeat nystagmus, and hypothyroidism [66,67,68]. In acute mania, Li doses can reach 1.2 mEq/L. Li is toxic when its concentration exceeds 1.5 mEq/L and can be lethal above 3.5 mEq/L [69,70]. The main side effects of Li are dose-dependent; thus, therapeutic doses should be employed to reduce any side effects [71,72,73,74]. Retrospective studies showed lower cancer incidence in psychiatric patients treated with Li therapy for bipolar disorder than in a control group not receiving Li therapy [75,76], which was suggested to be due to Li controlling cancer cell growth via inhibiting GSK-3β [77]. Interestingly, Li can inhibit 17 human magnesium-dependent phosphate transfer enzymes, which represents another potential anticancer mechanism of Li. Meanwhile, system biology studies demonstrated that 13 KEGG (Kyoto Encyclopedia of Genes and Genomes) pathway categories are most statistically enriched in the 265 genes that interact directly with GSK-3β. Given that the Li therapy impact is mostly systemic and the ability of Li to inhibit cancer cell growth has been shown, Li may also inhibit metastasis [54,76,78]. Li monotherapy (or in combination) was effective in inducing cancer cell apoptosis in breast cancer [79,80,81], colon cancer [82,83,84,85,86], esophageal cancer [82,87], glioblastoma [88], ovarian cancer [89], pancreatic cancer [88,90], prostate cancer [91,92,93], and thyroid cancer [94,95,96,97]. In vitro studies showed that Li induces apoptosis in lung cancer cell lines [98], and changes mRNA in leukemia [99,100] and lymphoma [101,102]. Given these studies, Li may have an anticancer effect through the induction of apoptosis and autophagy [103,104,105,106]. Li-induced autophagy may have other therapeutic applications beyond cancer treatment and regenerative medicine, such as treating autoimmune diseases [107,108].

**Figure 2:**
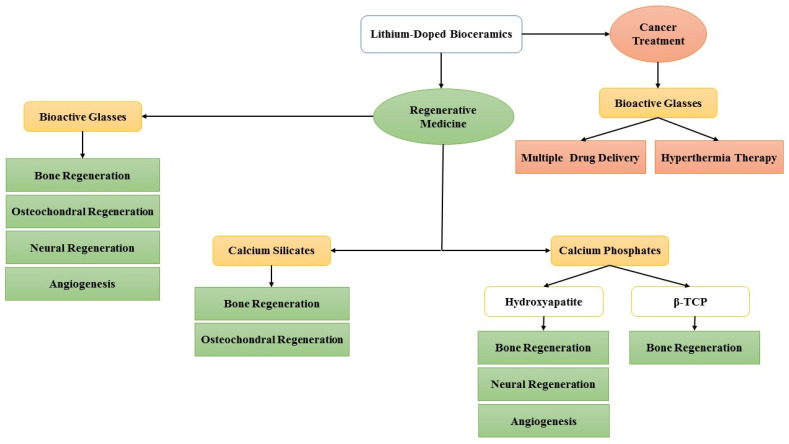
Current trends of research on the application of Li-doped bioceramics.

This review discusses the biological effects of Li ions and prospects for the application of Li-doped bioactive ceramics (Li-doped bioceramics) in regenerative medicine (abbreviations used throughout the article are summarized in Table 1). Current research trends in applications of Li-doped bioceramics are summarized in Figure 2; the bioceramics that have attracted most attention are BGs; bone and osteochondral regeneration are the predominant focus of the existing research, and despite the great potential of lithium-doped bioceramics for cancer treatment, wound healing, and nerve regeneration, these are comparatively nascent in their development.

## 2. Lithium and Its Biological Effects

### 2.1. Lithium and Stem Cell Fate

Li affects stem cells in various ways, mainly related to the inhibition of glycogen synthase kinase-3 beta (GSK-3β) and the activation of other pathways, such as activator protein-1 (AP-1), cyclic adenosine monophosphate (cAMP) response element binding protein (CREB), mitogen activated protein kinase (MAPK), WNT, and β-catenin signals. WNT and MAP kinase activation have a significant role in stem cell proliferation, wound healing, neural, and bone regeneration. Hence, Li affects stem cell fate, such as differentiation, proliferation, and regeneration. The neuroprotective and anti-inflammatory effects of Li are mainly related to GSK-3β inhibition, the deterrence of the pro-inflammatory cytokine response, and the production of reactive oxygen species (ROS), and they are stimulated by polymicrobial sepsis [109,110,111,112,113].

Angiogenesis-related gene expression can be attributed to the crosstalk between the canonical WNT and hypoxia-inducible factor 1-alpha (HIF-1α) signaling pathway [114]. The enhancement in vascular endothelial growth factor (VEGF) expression was observed through phosphatidylinositol 3 kinase (PI3-K)/(GSK-3β)-dependent and independent pathways in the brain endothelium and astrocytes, respectively, in the presence of Li [115]. Activating the WNT/β-catenin signaling impelled proliferation, survival, and migration, which are normal procedures in angiogenesis. These features were observed in vitro in human microvascular endothelial cells with LiCl [116]. A study demonstrated that Li improved self-renewal, stem cell homing, ability to build colonies, and self-renewal of hematopoietic stem cells (HSCs). Li can positively affect the maintenance and proliferation of mesenchymal stem cells (MSCs) [117], and Li impacts stem cells fate by enhancing autophagy, which has a crucial role in tissue development, remodeling, and regeneration [103,118,119].

### 2.2. Lithium and Osteogenesis

The mechanical properties of bone are of fundamental importance to its biological role [120,121] and a key property of tissue scaffolds for bone tissue engineering [122,123]. Li boosts β-catenin signaling, which stimulates bone growth in reply to mechanical load. In expanding sutures, Li enhances cell proliferation and β-catenin expression; the initial retardation in the differentiation of osteoprogenitors cells into mature osteoblasts by Li therapy was associated with the development of preosteoblasts, which pave the way for the enhancement of new bone regeneration in the vicinity of sutures. β-catenin influences osteoprogenitors proliferation and osteoblast maturation during mid-palatal suture osteogenesis. Li enhances β-catenin expression, boosting bone repair. Therefore, Li may boost the durability of orthodontic therapies, such as rapid palatal dilatation [124]. Evaluating the impact of GSK-3β deficiency in the mice model demonstrated that in vivo bone healing can be accelerated by GSK-3β inhibition; moreover, the results may be attributed to the impact of the higher activity of WNT/β-catenin in deficient mice [125]. In a similar study, rats were treated daily with LiCl or NaCl from 7 days before socket extraction up to 14 days after surgery. New bone development after tooth extraction was 37.5% (control), 23.8% (continuously treated), 53.9% (post-treated), and 63.2% (pre-treated) groups. Before or after socket extraction, Li enhances bone healing, and tooth removal during Li therapy may slow bone repair [126].

In a study on rats, it was observed that the administration of Li carbonate (45 mg/kg/day) caused bone deterioration in sexually mature healthy rats [127]. Another study reported that Li chloride (LiCl) debilitates BMP-2 signaling and creates a hindrance for osteogenic differentiation through an independent novel GSK-3β/WNT during the early stages of osteogenic differentiation [128]. In contrast to this study, others showed improving bone regeneration by the administration of Li, for example, it was reported that proliferation and osteogenic differentiation were enhanced at 4 mM and 10–12 mM of LiCl, respectively [129]. An important aspect of bone tissue is its mechanical properties and its ability to withstand forces exerted on it [120,121]. The effect of a 28-day Li therapy (140 mg/kg/day) on the mechanical properties of the bones of estrogen-deprived rats was investigated and, although a remarkable increase in the mechanical properties of cancellous bone was observed, increases in the mechanical properties of compact bone were small; this suggests that the use of Li in improving the mechanical properties of bone holds promise for long-term clinical applications [130]. A comparable study revealed that 150 mg/kg/2 days of LiCl could enhance bone regeneration substantially in osteoporotic mice. Higher bone volume, trabecular thickness, trabecular number, and osseointegration were assessed with Micro-CT, and the maximum push-out force (N) and implant−bone contact shear strength (N/mm^2^) were stronger in the LiCl group (36 ± 6 N vs. 105 ± 12 N, 1.9 ± 0.4 vs. 5.6 ± 0.7 N/mm^2^, respectively) [131]. The effects of systemic LiCl administration on the socket healing of estrogen-deficient rats were evaluated, finding that LiCl improved bone regeneration in rats with estrogen deficiency, especially in the initial healing [132]. Bone regeneration by Li treatment is therefore dose-dependent and the dosage may be dependent on the stem cell source [133].

Li acts via hedgehog pathways (Hh). By simultaneously impacting the Hh and WNT pathways, LiCl diminishes adipogenesis and improves osteogenesis in bone marrow mesenchymal stem cells (BMSCs) [134]. The adipogenic gene (CEBPA, CMKLR1, and HSD11B1) expression of human mesenchymal stem cells (hMSCs) after exposure to Li was reduced, while the expression of alkaline phosphatase (ALP), Runt-related transcription factor 2 (Runx2), bone sialoprotein (BSP), and collagen 1 synthesis were elevated [135]. A key clinical challenge is fracture healing, which can be a lengthy process and fails in 5–10% of cases. Femoral fracture in rats was used as a model to optimize Li administration variables (such as onset time, curing duration, and dose); administrating a low dose of Li (20 mg/kg) for two weeks after fracture revealed the most promising results [136]. Furthermore, another effect of Li on bone regeneration is increasing bone mineral density after the administration of Li. Dual-energy X-ray absorptiometry (DEXA) at the lumbar spine and hip in 75 normal participants and 75 Li-treated patients showed that bone density was raised by 5.3% at the femoral neck, 7.5% at the trochanter, and 4.5% at the spine. Li-treated patients had reduced ALP, osteocalcin, and serum CTX [137].

The immune system plays a significant role in bone regeneration. Osteoimmunomodulation (OIM) is an area of focus that has been developed to study the immune response during osteogenesis [138,139]. Li modulates immune cells, especially macrophages through chemokine gene expression [140]. Osteal macrophages (osteomacs), especially CD169+ osteomacs pro-anabolic support contribute to osteoblasts during bone hemostasis and regeneration [141]. In vitro studies revealed that Li reprograms macrophages to the M2 phenotype, leading to improvement in osteogenic differentiation in rat BMSCs. Additionally, LiCl prevents p38mitogen-activated protein kinase (p38MAPK) and extracellular signal-regulated kinase (ERK) from phosphorylation. Hence, it accelerates bone regeneration, for which these studies help to find new treatments wearing debris-induced osteolysis [142,143].

**Figure 3:**
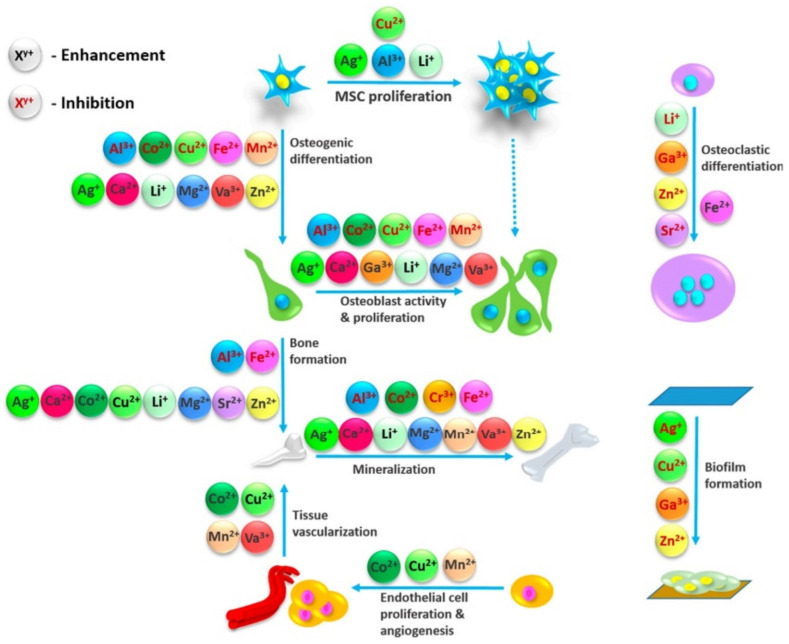
Influence of metal ions on the variety of processes involved in bone regeneration. Reproduced from [145] with permission (open access, Attribution 4.0 International (CC BY 4.0)).

The results of computerized tomography and bone histomorphometry showed that the local Li_2_CO_3_ administration can accelerate bone healing in rat tibia defective lesions by raising lamellar bone ratios versus controls, and the acceleration in the recovery of bone damage through boosting osteoblastogenesis and preventing osteoclastogenesis was achieved effectively by the local delivery of Li [144]. However, decreasing immune-responsive genes (CXCL1, CXCL12, CCL20, IL7, and IL8) and osteoclastogenic factors were reported before [135]; therefore, increasing bone mineral density can result from inhibiting osteoclastogenesis caused by Li. The crucial roles of Li in different stages of bone regeneration is summarized in Figure 3 [145].

### 2.3. Lithium and Bone and Cartilage Regeneration

The majority of the work on Li focuses on bone regeneration. Given the similarity in the development of bone and cartilage, Li may also induce cartilage regeneration. An in vitro study showed the growth of cartilage on LiCl-polydopamine (PDA)-coated 3D-printed poly-ε-caprolactone (PCL)-based scaffolds, and glycosaminoglycan (GAG) production was increased as was chondrogenic marker gene expression [146]. Teeth are another hard tissue and activating WNT/β-catenin signaling affects the rate of dentin secretion and cementoblastic differentiation [147,148,149,150]. An in vivo study in a rat pulp capping model showed that the local administration of LiCl leads to the induction of compensatory dentin formation through WNT/β-catenin signaling [151]. Another study reported that the WNT signaling pathway is crucial in regulating dentin sialophosphoprotein (Dspp) expression, and LiCl promotes mRNA levels of Axin2, Kallikrein 4 (Klk4), and Dspp while attenuating the expression of osteopontin. Therefore, using LiCl as a capping-material for dentine regeneration may be promising [152]. Since Li effects are dose-dependent, the overuse of Li can result in severe dental putrefaction and deterioration in the tooth structure, which is linked with dentin mineral loss [153]. Therefore, using Li in dentistry still requires extensive studies [154].

### 2.4. Lithium and Wound Healing

Li activates the WNT pathway; WNT7a has a crucial role in wound healing, especially regenerating damaged vessels and diminishing the inflammatory response in diabetic wounds (with or without obesity) and epithelial differentiation [155]. The size of the wound is regulated by canonical WNT/β-catenin signaling, which also mediates the role of transforming growth factor beta (TGF-β) in cutaneous healing [156,157,158]. Therefore, Li-ions are a suitable target for wound healing. In live animals, initiating the WNT signaling pathway by employing a pump specialized for negative pressure wound therapy (NPWT) and LiCl promoted the migration of cells into simulated wound sites. The minimum LiCl demanded to fill the simulated wound is 10 mM [159]. Similar results were achieved by loading LiCl in chitosan hydrogel wound dressings in male C57BL/c mice [160].

**Figure 4:**
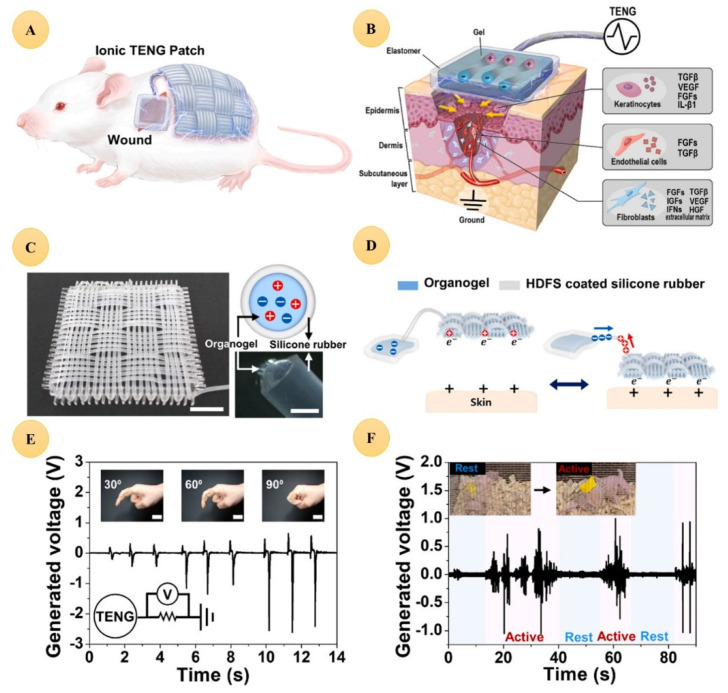
An ionic TENG (iTENG) patch and its potential wound-healing applications. (**A**) Schematic illustration of an iTENG patch for accelerated wound healing based on tribotronics. (**B**) Schematic depiction of accelerated wound healing due to the secretion of biological molecules and the formation of new cutaneous tissue under a self-powered EF driven by an iTENG patch. (**C**) Optical image of the ionic fabric (scale bar: 1 cm. Inset: Schematic diagram of a cross-section of the ionic fabric and magnified image of a fiber.) Conductive organogel is injected into an HDFS-treated silicone tube (scale bar: 500 μm). (**D**) Schematic illustration of the biomechanical energy harvesting mechanism of the iTENG, which relies on friction between the iTENG and skin. (**E**) Voltage generated upon bending at 30°, 60°, and 90° (scale bar: 3 cm. Inset: Optical image of wearable ionic fabric on a bent index finger). (**F**) Voltage generation of a self-motion-driven iTENG patch applied to the back of a BALB/c nude mouse. Reproduced from [163] with permission.

One of the most important applications of Li is in energy storage as Li-ion batteries, which are the most promising electrochemical energy storage devices [161]. Electrotherapy creates new opportunities in wound healing [162], and wearable ionic triboelectric nanogenerator (iTENG) patches (created from a stretchable platform based on LiCl-loaded organogels and elastomeric microtubular structures) utilized the therapeutic effects of Li-ions in wound healing, and moreover contributed to creating and transmitting electrical stimulation (Figure 4) [163], which is very promising in wound healing applications [164].

### 2.5. Lithium and Nerve Regeneration

Li was shown to promote the proliferation of progenitor cells in the hippocampus’s dentate gyrus and to boost the mitosis of Schwann cells; neurogenesis is connected to Li’s neuroprotective and neurotrophic effects, synaptic plasticity improvement, cell survival enhancement, and apoptosis reduction [117]. GSK-3β inhibitors, especially mood stabilizers such as LiCl, could be neuroprotective or anti-inflammatory agents. Li can enhance remyelination by boosting the expression of MPZ and PMP22 promoter activity, as well as transcripts, and protein levels. LiCl promotes myelin gene expression, maintains myelin integrity, and catalyzes the recovery of mouse’ whisker movements following facial nerve compression injury; it also promotes the remyelination of sciatic nerves. Moreover, the mechanism of LiCl interaction with Schwann cells can be attributed to raising the amount of β-catenin and provoking its nuclear localization [165].

The hypothesized neuroprotective effects of Li include the inactivation of *N*-methyl-D-aspartate receptors, the activation of PI3-K/Akt cell survival pathway, boosting expression of cytoprotective Bcl-2, and the suppression of GSK-3β [166]. Schwann cell viability and proliferation rates were increased at 5, 10, 15, and 30 mM LiCl. Wound healing was due to suppressing the migration of Schwann cells [167]. Thus, in peripheral nerves, Li improved remyelination by enhancing the expression of peripheral myelin genes, resulting in their proliferation and attenuating the migration of Schwann cells. In addition to peripheral nerves, Li has a significant neuroprotective role in central nervous system (CNS). For example, it has been reported that Li attenuates neuronal damage after acute spinal cord injury (SCI) and promotes neurological recovery by inducing autophagy [168]. Brachial plexus damage is one of the most common spinal cord injuries that often involves intense root avulsion resulting in the permanent paralysis of the innervated muscles. The impaired regeneration of motoneurons from the spinal to the peripheral nerve system (PNS) is one of the leading causes of inadequate treatment. By inhibiting GSK-3, Li therapy can improve motoneuron regeneration from the CNS to the PNS [169]. The outcomes of daily intraperitoneal LiCl administration after a 20-week rehabilitation on the immediate reimplantation and avulsion of the C7 and C8 ventral roots were studied, and Li along with reimplantation permitted 45.1% of motoneurons to be rescued from the injury as well as improving the quantity and median diameter of nerve fibers [170]. Another study demonstrated that Li, started during the early remyelination phase, preserved it, despite the late stage of the process [171]. Locally releasing Li from hyaluronic acid in a silicon conduit on a rat sciatic nerve injury was observed to increase nerve regeneration in rats [172].

### 2.6. Lithium and Antibacterial and Antiviral Activities

The antibacterial and antiviral activities of biomaterials are beneficial because infection and the presence of pathogens are among the most critical problems that inhibit tissue regeneration [173,174,175,176], and moreover, it is recognized that bacteria and viruses are recognized to have the potential to induce carcinogenesis [177,178,179,180]. The main metal-mediated antibacterial mechanisms are membrane disruption, ROS generation, macrophage activation, and protein/DNA damage [181,182]. In the case of Li, immunostimulating, anti-prostaglandin actions, inhibiting viral replication, and reducing lymphopenia are reported as being the primary antibacterial and antiviral mechanisms [183,184]. Moreover, Li affects autophagy, and it has been reported that autophagy has a vital role in virally infected cells; hence, further studies to investigate the antiviral mechanism of Li are important [103,185].

**Figure 5:**
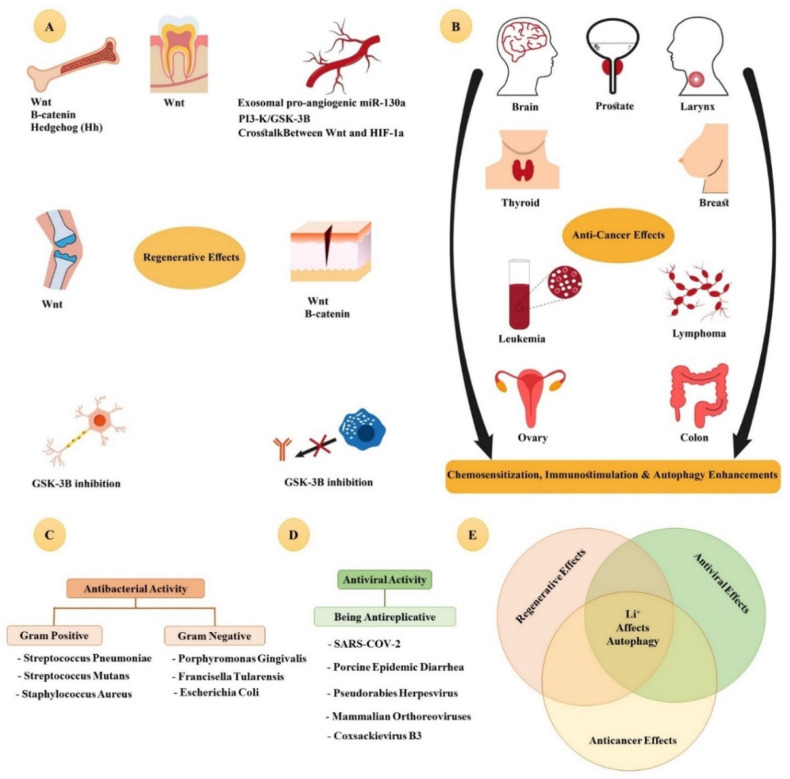
An overview of the biological effects of lithium. (**A**) Regenerative effects and their main signaling pathways that are activated in each tissue. Lithium can cause regeneration in several tissues, including bone, cartilage, dentin, nerve, skin, and vascular system. A WNT/β-catenin signaling pathway is the primary signaling pathway activated by lithium. Additionally, it has an anti-inflammatory response by inhibiting the GSK-3β pathway. (**B**) The anticancer effects of lithium in several types of prevalent cancers with a high mortality rate, including pancreatic cancer, thyroid cancer, esophageal cancer, colon cancer, prostate cancer, ovarian cancer, breast cancer, lung cancer, leukemia, and glioblastoma. Lithium has shown an anticancer effect and has been used as a singular or adjunct treatment. Hence, lithium can be considered a chemosensitizer in chemotherapy. (**C**) Antibacterial properties. Lithium has antibacterial properties against both Gram-positive and Gram-negative bacteria. (**D**) Lithium has antireplicative effects against several types of viruses. (**E**) The crucial point of the biological properties of lithium, including its antiviral, anticancer, and regenerative effects, is the effect of lithium on autophagy.

Given the impact of SARS-CoV-2 in global health, various anti-inflammatory and antiviral treatments are under investigation, including Li [186,187,188,189,190,191,192]. To date, the antiviral activity of Li in different viruses (including porcine epidemic diarrhea, pseudorabies herpesvirus, Orthoreoviruses, and Coxsackievirus B3 virus) has been proved [193,194,195,196]. Additionally, its antibacterial activity against Gram-negative bacteria (including *Porphyromonas gingivalis*, *Francisella tularensis*, *Aggregatibacter actinomycetemcomitans*, *Klebsiella pneumoniae*, *Escherichia coli*, *Burkholderia pseudomallei*, and *Pseudomonas aeruginosa*) and Gram-positive bacteria (including *Streptococcus pneumoniae*, *Streptococcus mutans*, and *Staphylococcus aureus*) has been reported [197,198,199,200,201,202]. A summary of the biological effects of Li and the relation between these effects and autophagy is presented in Figure 5.

## 3. Lithium-Doped Bioceramics in Regenerative Medicine

Bioceramics can be used in both hard and soft tissue regeneration. The three main groups of bioceramics that incorporate Li-ions in their structure include bioactive glass (BG), calcium phosphates (including hydroxyapatite (HA) and beta-three calcium phosphate (β-TCP)), and silicates.

### 3.1. Lithium-Doped Bioactive Glasses (Li-BGs)

Bioactive glasses (BGs) are bioceramics promoting hard tissue regeneration by creating a layer of HA on their surfaces [203,204]. An advantage of BGs is the inclusion of various ions to their structure to improve their performance. In recent years, one of the ions added to the structure of BG is Li; Li-BG is commonly used in bone regeneration and osteochondral repair [205]. For instance, a study investigated the biocompatibility and bioactivity of 45S5 Li-BG that was prepared by a melt quenching method. Li in low concentrations inhibited apatite formation, resulting in the compactness of the structure [206]. Additionally, Li-substituted bioglasses caused increasing ALP activity and cell proliferation in a dose-dependent manner, reducing the rate of ion release and apatite formation [207]. Li_2_O contents within the therapeutic range (below 8.3 ppm) have been reported, which should be between 2.5 and 5 wt% in Li silicates, 45S5 Li-BG, and their scaffolds. Li-BGs generally crystallize into the phases Li_6_P_6_O_18_ and Li_3_PO_4_, as well as combeite (Na_2_Ca_2_Si_3_O_9_) and silicorhenanite (SiO_4_(PO_4_)_2_Ca_5_) [208]. Investigating the effect of Li precursors on the structure–property relationships of Li–silicate sol–gel BG revealed that nitrate, in comparison to citrate, has a higher affinity for Li. In contrast, citrate has a lower decomposition temperature that is advantageous [209].

One of the areas in which bioceramics have a wide range of applications is dentistry [210]. A new class of glass–ceramics based on Li_2_O-SiO_2_ called Li disilicate (LD) was conceived for dentistry due to its aesthetics, chemical durability, high fracture strength, and inertness in the buccal environment [211,212]. LD bioactivity begins after 14 days, and after 21 days, a mineralized matrix develops from a demineralized matrix [213]. Similar results were reported in a case report for zirconia-reinforced Li silicate (ZLS) [214]. The remineralization process induced by 45S5 Li-BG containing 5-wt% of Li and its great antibacterial activity is prevalent in oral diseases [202].

BGs are used in bone regeneration due to release crucial ions [215]. The release of Li from different 45S5 BGs, which is designed for bone regeneration, has been explored by Da Silva et al. [216]. Similar to LiCl treatment, local Li-ion release upregulated WNT pathway expression in 17IA4 cells. However, high concentrations of BG may cause cytotoxicity due to changes in the pH of the solution. Compared to Li-doped phosphate-based bioactive glasses (Li-PBGs), Li-doped borate-based bioactive glasses (Li-BBGs) release Li at a slightly higher rate and amount. The quantitative polymerase chain reaction (qPCR) analysis of AXIN2 expression found that Li-BBGs had a higher gene expression. Li-BBGs release more Li, explaining these results [217].

The synergistic effect of dopant ions and the ease of preparing polymer composites are two significant advantages of BGs. For example, the use of Sr-doped BGs for bone regeneration is well established [218]. The impact of single and binary strontium and Li doping on BG scaffolds in vivo has been investigated employing histochemical and micro-computed tomography (micro-CT) analysis of a femoral defect of rabbits as a model at 2, 4, and 6 months. Li-doped scaffolds have mild bone regeneration, while Sr and Li + Sr-doped scaffolds had excellent osseous tissue formation. Moreover, micro-CT data showed that Li + Sr samples have the highest degree of vascularity, peripheral cancellous tissue formation, and cortical tissue inside implanted samples. Thus, doping Sr and Li to BG can improve bone regeneration, especially in early in vivo osseointegration [219]. Nanobiocomposite scaffolds consisting of Li-doped mesoporous bioactive glass (Li-MBG) and a block copolymer (mPEG-PLGA-b-PLL) were observed to significantly improve MC3T3-E1 cell proliferation, attachment, and ALP activity [220]. Thus, doping Li and other beneficial ions for bone regeneration to BGs such as Sr may synergistically enhance its regenerative effect. They may also be used to prepare polymeric nanocomposite scaffolds.

The majority of BGs’ regenerative applications are orthopedic. Osteochondral lesions are common worldwide and pose significant treatment challenges for orthopedic specialists due to unsatisfactory treatments [221]. Although regenerative medicine has proposed new treatment strategies, such as matrix-associated autologous chondrocyte implantation (MACI), layered scaffolds in acellular or cellular approaches for use in the clinic [222], the most challenging task is to create biomaterials that can regenerate both bone and cartilage. These requirements make this field difficult [223]. Gradient scaffolds for osteochondral tissue engineering are exciting, but designing them for clinical application is challenging [224]. Because of these reasons, simplifying biomaterial system design is critical. Li-releasing BGs derived from sol-gel processes are suitable for cartilage regeneration [146,225]. Thus, using Li-doped BGs for osteochondral tissue engineering is promising but not well investigated. For example, Li-MBG was used in a rabbit osteochondral defect study. After 8–16 weeks of implantation in osteochondral defects, Li-MBG scaffolds outperformed pure MBG scaffolds in terms of the regeneration of subchondral bone and hyaline cartilage-like tissues, suggesting Li-doped BGs have great potential in osteochondral regeneration [226].

Although bioceramics and BGs appear limited to orthopedics and hard tissue reconstruction, these materials have found many applications in soft tissue reconstruction due to one of their pro-angiogenesis properties. The lack of mature and functional vasculature has severely hampered the clinical translation of tissue-engineered constructs [227]. As stated previously, Li-ions can induce angiogenesis, making Li-doped BGs promising materials for improving angiogenesis [114,116]. Exposure to 45S5.5 Li-BG ionic dissolution products improved angiogenesis by increasing integrin αvβ3 subunit β3 expression and vascular density in quail embryo CAMs. The ionic dissolution products of 45S5.5 Li-doped BGs can be considered inorganic angiogenic agents, which can be used in place of expensive and potentially harmful growth factors [205,228,229]. It has also been claimed that Si and Li-ions have synergistic effects on the activation of the WNT/β-catenin canonical pathway and the production of proangiogenic cytokines (insulin growth factor 1 (IGF1) and TGF- β) [205,230]. A separate study demonstrated that 45S5 Li-BG could improve human umbilical vein endothelial cells’ (HUVECs) pro-angiogenic ability by downregulating PTEN protein and activating the AKT pathway, which increases endothelial cell proliferation, migration, and tube formation and enhances the expression of pro-angiogenic genes [231]. Therefore, it appears that adding Li-ions to BGs can stimulate angiogenesis, though more research is required to fully grasp the mechanism.

Finally, given Li’s neuroprotective and neurogenic properties and the use of BGs in neural regeneration, Li-doped BGs may be helpful in neural tissue engineering scaffolds [232,233]. Despite the several neuroprotective advantages of Li, there are relatively few studies related to Li-doped bioceramics for neural regeneration. Extensive burns, for example, can cause nerve damage, and different Li_2_O contents have been added to BGs to support nerve healing and angiogenesis. In the proper dilution ratio, Li-BG extracts advanced the proliferation of Schwann cells and HUVECs. Li-BG extracts with adequate Li- and Si-ions promoted Schwann cell migration [234]. A new strategy for neural regeneration may be Li-BGs and their polymeric composites.

### 3.2. Lithium-Doped Calcium Phosphates

Calcium phosphates, including HA and β-TCP, have always been considered the first bioceramics used in regenerative medicine due to their extracellular matrix nature. HA, which is found in the body, is one of the bioceramics whose function is improved by adding ions such as Li [235]. For example, improving fracture bone healing with Li-doped calcium phosphate cement (Li/CPC) has been studied; Li/CPC extracts can stimulate the in vitro proliferation and differentiation of osteoblasts by releasing Li-ions (Li^+^) at 25.35–50.74 mg/L via the WNT/β-catenin pathway. The effects of the local Li^+^ release in rat tibia defects were also studied in vivo using CPC and Li/CPC. Compared to CPC, Li/CPC showed better osteoconductivity, osteogenesis, and osseointegration by increasing bone mass and promoting defect repair [236]. Calcium phosphate/Li coatings improved MG63 cell attachment, early proliferation, and biocompatibility [237], so doping Li-ion to calcium phosphate can improve its potential biomedical application. For example, the glucocorticoid-induced osteonecrosis of the femoral head (GIONFH) affects young people and middle-aged adults, and to treat GIONFH, a composite scaffold with Li as a WNT signal activator and erythrogenin (EPO) to upregulate the HIF-1/VEGF pathway was designed. To this end, a porous gelatin/Li-doped-hydroxyapatite nanoparticles/gelatin microspheres/rhEPO (Li-nHA/GMs/rhEPO) composite scaffold was created. The in vitro results showed increased osteogenic and angiogenic factors and activating factors in the WNT and HIF-1/VEGF pathways. Additionally, in the GIONFH rabbit model, this scaffold improved new bone formation and repaired femoral head defects [238]. Consequently, more research on Li-doped HA and its nanocomposites in treating GIONFH or similar conditions seems logical.

Orthopedic implants are another possible application for Li-doped HA. In simulated body fluid (SBF), Li-HA scaffolds were hydrolyzed, and they can also be degraded by cells. Li-HA scaffolds increased PO_4_^3−^ release in a degradation medium, which increased osteoblast physiological activity and sped up Li-HA degradation. The addition of Li to HA also increased its compressive strength. Moreover, SEM and MTT assays showed that the degradation products of Li-HA scaffolds aided osteoblast proliferation [239]. Although adding Li to HA did not affect the degradation rate, doping Li into HA scaffolds increased new bone formation by decreasing GSK- 3β and β-catenin mechanisms, but did not have a significant angiogenic effect [240]. The incorporation of Li in HA causes densification [241], with greater crystallinity for Li-doped HA than undoped HA. Li also reduces the dielectric constant, which is good for dental and orthopedic applications [242]. A study evaluated the physical, mechanical, and biological properties of Li-doped calcium phosphates, which showed the growth of an apatite layer in SBF [243]. Metallic implants coated with Li-HA thin films have been studied. FTIR spectra revealed the coatings’ high biomineralization potential; Li_3_PO_4_ and Li_2_CO_3_ as doping reagents were observed to increase the growth of hMSCs on film surfaces, suggesting Li-doped bio-derived materials as a promising next-generation coated implant material with rapid osteointegration [244].

Seeding stem cells on the new generation of implants may improve clinical applications. For instance, simultaneous nerve and bone tissue regeneration in spinal cord injuries are required (SCI). Hydroxyapatites are bioresorbable materials with good biocompatibility and osteoconductivity, so their use in spinal surgery is possible. The theranostic agent nanocrystalline calcium HAs, incorporating Li^+^ (Li-nHA) doped with europium (Eu^3+^), have excellent potential for treating SCI. Human olfactory ensheathing cells (hOECs) and adipose tissue-derived multipotent stromal cells were used to assess the biocompatibility of the nanoparticles. The results show a promising approach to SCI treatment using regenerative strategies [245]. A Li-HA porous scaffold seeded with hypoxia-preconditioned BMSCs for bone regeneration was also evaluated in vitro and in vivo. The data revealed that 1.5%Li-HA had the best in vitro cell proliferation and bone formation, with a decrease in GSK-3β and an increase in β-catenin, though Li did not affect angiogenesis significantly. Hypoxia-preconditioned BMMSCs improved angiogenesis and osteogenesis by activating the WNT and HIF-1 signal pathways [246]. A new strategy for improving the clinical efficacy of Li-doped HA scaffolds seeded with stem cells appears to be emerging.

Β-TCP scaffolds based on excellent biocompatibility and compositional similarity to the natural bone have received attention as ceramic implants for bone repair and augmentation. However, the high solubility of β-TCP may cause refracture due to implant degradation and inflammatory reactions [247]. Li-doping β-TCP increases dissolution and thermal stability [248,249], so doping β-TCP with Li-ions appears to have potential to improve clinical applications.

### 3.3. Other Lithium-Doped Bioceramics

Calcium silicate bioceramics are widely applied in tissue engineering and drug delivery [250,251,252,253]. The apatite mineralization of a bioactive composite based on poly (dopamine) (PDA) and Li-doped silica nanospheres (LSNs) coated on polyetheretherketone (PEEK) [254] surfaces (LSN-PDA-PEEK) in SBF was evaluated, and the bioactivity was observed to be higher than that of neat PDA coated on PEEK (PDA-PEEK) and PEEK. LSN-PDA-PEEK also stimulated rBMSC responses more than PDA-PEEK and PEEK. Moreover, in vivo, LSN-PDA-PEEK increased bone tissue responses compared to PDA-PEEK and PEEK [255]. Adding 5% Li to mesoporous silica nanospheres (MSNs) increases their degradability. Moreover, Li-doped mesoporous silica nanospheres (LMSNs) had more significant stimulatory effects on BMSC attachment and proliferation than MSNs due to Li-ion release. LMSNs may also improve BMSC ALP activity and the expression of osteogenesis-related genes (osteopontin (OPN), osteocalcin (OCN), Runx2, and ALP). Thus, LMSNs have a potential application in bone regeneration [256].

**Figure 6:**
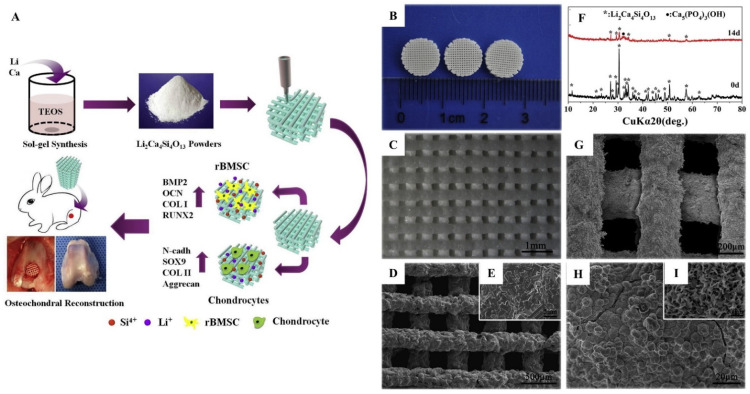
(**A**) Schematic illustration of the application of Li_2_Ca_4_Si_4_O_13_ scaffolds for osteochondral reconstruction. Pure-phase Li_2_Ca_4_Si_4_O_13_ powders were successfully synthesized by the sol–gel method. Three-dimensional-printed Li_2_Ca_4_Si_4_O_13_ scaffolds not only promoted cartilage maturation, but also stimulated osteogenic differentiation in vitro. On the other hand, Li_2_Ca_4_Si_4_O_13_ scaffolds significantly accelerated cartilage regeneration as well as promoting subchondral bone reconstruction in vivo. (**B**–**I**) Surface morphology and XRD analysis of Li_2_Ca_4_Si_4_O_13_ scaffolds. Digital photograph (**B**), optical microscope image (**C**), and SEM images (**D**,**E**) of 3D-printed Li_2_Ca_4_Si_4_O_13_ scaffolds. The prepared porous Li_2_Ca_4_Si_4_O_13_ scaffolds possessed a controlled pore size (~250 μm). XRD analysis (**F**) of Li_2_Ca_4_Si_4_O_13_ scaffolds before/after soaking in the simulated body fluids for 14 days, and SEM images (**G**–**I**) of Li_2_Ca_4_Si_4_O_13_ scaffolds after soaking in the simulated body fluids for 14 days. Li_2_Ca_4_Si_4_O_13_ scaffolds induced distinct apatite mineralization on their surface. Reproduced from [258] with permission.

As a result of the synergistic interaction of Li and Si-ions, Li-ions can enhance the biological effectivity of calcium silicates. This synergistic effect can be seen in osteochondral regeneration. Osteoarthritis (OA) causes cartilage lesions that spread to the subchondral bone. The regeneration of both tissues is required to repair osteochondral OA defects. Extracts of biomaterials containing Li and silicon have significantly increased chondrocyte proliferation and maturation, and favored the osteogenic differentiation of rabbit mesenchymal stem cells (rBMSCs). A histological and micro-CT analysis revealed that Li-doped calcium silicate (LCS) scaffolds promoted osteochondral regeneration in vivo; Li- and Si-ions released from LCS scaffolds are important in osteochondral regeneration, suggesting that LCS scaffolds are promising biomaterials for osteochondral repair [257]. In a similar study, pure phase LCS (Li_2_Ca_4_Si_4_O_13_ and L_2_C_4_S_4_) scaffolds were synthesized by the sol–gel method and then 3D printed. These scaffolds have controlled biodegradability and good apatite mineralization capacity. The ionic products of L_2_C_4_S_4_ also significantly increased chondrocyte proliferation and maturation and rBMSC osteogenic differentiation. In osteochondral defects of the rabbit, the L_2_C_4_S_4_ scaffolding favored both cartilage and subchondral bone regeneration (Figure 6) [258]. Three-dimensional-printed LCS and its composites offer opportunities to generate scaffolds to treat difficult-to-treat disease (such as OA) conditions (particularly as the printing process facilitates the inclusion of macropores for vascularization). Another study evaluated the in vitro and in vivo osteogenic properties of 3D-printed lithium magnesium phosphate (Li_0_._5_Mg_2_._75_ (PO_4_)_2_, Li_1_ Mg_2_._5_ (PO_4_)_2_, and Li_2_Mg_2_ (PO_4_)_2_) prepared by the sol–gel method. Interestingly, the lithium magnesium phosphate has a lower porosity and higher compressive strength, and raises cellular proliferation, osteogenic differentiation, and proangiogenic activity; moreover, lithium magnesium phosphate significantly improved bone regeneration in critical-size calvarial defects of rats [259]. Recent developments in dental restorations used Li germanosilicate glass-ceramics doped with rare-earth oxides [260].

### 3.4. Lithium-Doped Bioceramics for Anticancer Applications

**Figure 7:**
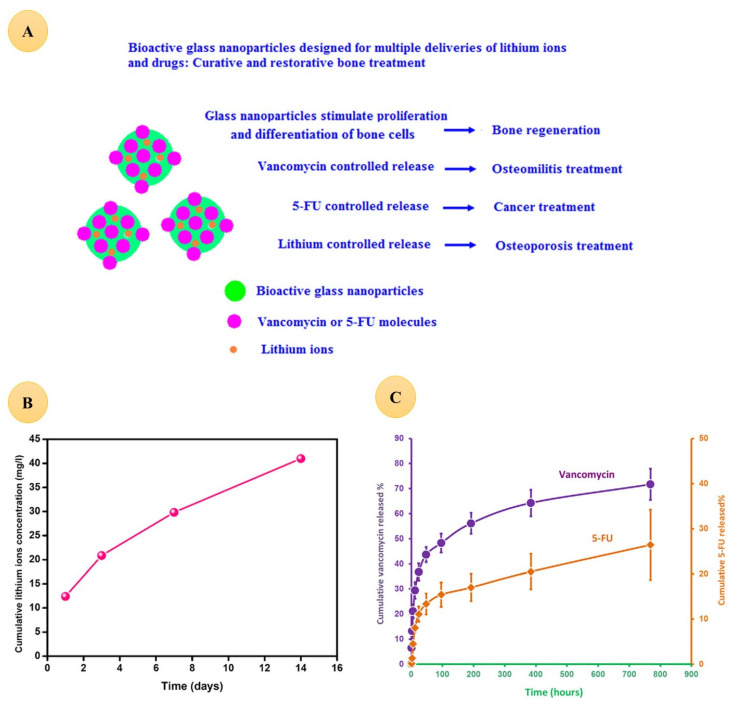
(**A**) Bioactive glass nanoparticles designed for multiple deliveries of lithium ions and drugs as curative and restorative bone treatment. (**B**) The cumulative concentration of released lithium ions shown against the soaking time. (**C**) The cumulative percentage of vancomycin and 5-FU released form glass nanoparticles against time. Reproduced from [263] with permission.

Drug delivery is an important potential application of bioceramics, especially nanobioceramics [261]. Additionally, as previously stated, Li has anticancer properties. Some researchers have found that using Li with other drugs has a synergistic effect on cancer cells and may be used for combinational therapy [88,262]. Li may cause tumor chemosensitization [106]. Thus, the use of Li-doped bioceramics could be an exciting research area. However, there are few reports in the literature exploiting these bioceramics for such applications. Li-BG nanoparticles loaded with vancomycin or Fluorouracil (5-FU) were designed to deliver Li-ions and drugs simultaneously to treat osteomyelitis, bone cancer, and osteoporosis; drugs are released via a diffusion-controlled process, and the release profile is dependent on the drug concentration applied in the loading stage (Figure 7) [263].

Li-ferrite BGs were prepared using the sol–gel method to assess their use in hyperthermia therapy, and these BGs can provide cancer hyperthermia up to 47.2 °C (however, they did not test their effects on a cancer cell line or compare the effects of presenting Li-ions on cell death) [264].

## 4. Conclusions

Li is widely used to store energy, particularly in batteries [265,266,267,268] and capacitors [269,270], and we foresee nanogenerators [271] (including stimuli-responsive nanogenerators, e.g., photoactivatable nanogenerators [272]) will have broad applications in medical fields, such as regenerative medicine, rehabilitation, and cancer treatment [273,274,275,276], particularly as nanogenerators have been shown to increase the efficacy of chemoimmunotherapy for non-small-cell lung cancer [277]. Therefore, Li-doped bioceramics may be good candidates for nanogenerators for advanced multifunctional systems in cancer treatments and regenerative medicine [272,278]. Li has anti-replication properties in viruses and is anti-mitotic in cancer cells, but it simultaneously stimulates stem cell proliferation, which may be an evolved regulatory system. However, more studies on the effects of Li on autophagy in cancer cells, virally infected cells, and stem cells and their related signaling are also necessary.

Li has a variety of biological properties that can be influential in stem cell therapy, in the development of the next generation of antibacterial, antiviral, and anticancer agents, as well as in tissue regeneration, and opportunities exist for fundamental studies to understand the role of Li in biological processes. Li is a widely used medication for various mental illnesses [279,280,281,282], and we foresee significant potential for further clinical applications of biomaterials incorporating Li in some manner (e.g., doped ceramics and gels), supported by the large number of ongoing clinical trials employing Li in some fashion (>3000 clinical trials in the Cochrane Central Register of Controlled Trials [283]). The purpose of this review was to provide to interested readers an overview of some of these clinical trials.

## Figures and Tables

**Table 1:** Abbreviations used in this review.

Abbreviation	Word or Phrase
ALP	Alkaline phosphatase
AP-1	Activator protein-1
BG	Bioactive glass
BMSC	Bone marrow mesenchymal stem cell
β-TCP	Beta-three calcium phosphate
cAMP	Cyclic adenosine monophosphate
CNS	Central nervous system
CREB	Response element binding protein
DEXA	Dual-energy X-ray absorptiometry
Dspp	Dentin sialophosphoprotein
EPO	Erythrogenin
ERK	Extracellular signal-regulated kinase
5-FU	Fluorouracil
GAG	Glycosaminoglycan
GIONFH	Glucocorticoid-induced osteonecrosis of the femoral head
GSK-3β	Glycogen synthase kinase-3 beta
HA	Hydroxyapatite
Hh	Hedgehog pathways
HIF-1α	Hypoxia-inducible factor 1-alpha
HSC	Hematopoietic stem cell
HUVEC	Human umbilical vein endothelial cell
IGF1	Insulin growth factor 1
iTENG	Ionic triboelectric nanogenerator
KEGG	Kyoto Encyclopedia of Genes and Genomes
Klk4	Axin2, Kallikrein 4
LCS	Lithium-doped calcium silicate
LD	Lithium disilicate
Li-BG	Lithium-doped bioactive glass
Li-BBG	Lithium-doped borate-based bioactive glass
Li-MBG	Lithium-doped mesoporous bioactive glass
LMNS	Lithium-doped mesoporous silica nanosphere
Li-nHA/GMs/rhEPO	Gelatin/lithium-doped-hydroxyapatite nanoparticles/gelatin microspheres/rhEPO
Li-PBG	Lithium-doped phosphate-based bioactive glass
LPPEEK	Lithium-doped silica nanospheres coated on polyetheretherketone surface
LSN	Lithium-doped silica nanosphere
MACI	Matrix-associated autologous chondrocyte implantation
MAPK	Mitogen-activated protein kinase
micro-CT	Micro-computed tomography
MSC	Mesenchymal stem cell
MSN	Mesoporous silica nanosphere
NPWT	Negative pressure wound therapy
OA	Osteoarthritis
OIM	Osteoimmunomodulation
p38MAPK	P38mitogen-activated protein kinase
PCL	Poly-ε-caprolactone
PDA	Polydopamine
PEEK	Polyetheretherketone
PI3-K	Phosphatidylinositol 3 kinase
PNS	Peripheral nerve system
qPCR	Quantitative polymerase chain reaction
rBMSC	Rabbit mesenchymal stem cell
ROS	Reactive oxygen species
Runx2	Runt-related transcription factor 2
SBF	Simulated body fluid
SCI	Spinal-cord injury
TGF- β	Transforming growth factor beta
VEGF	Vascular endothelial growth factor
ZLS	Zirconia-reinforced lithium silicate

## Data Availability

Not applicable.
